# A Wearable Extracorporeal CO_2_ Removal System with a Closed-Loop Feedback

**DOI:** 10.3390/bioengineering11100969

**Published:** 2024-09-27

**Authors:** Andrew Zhang, Brian J. Haimowitz, Kartik Tharwani, Alvaro Rojas-Peña, Robert H. Bartlett, Joseph A. Potkay

**Affiliations:** 1 Extracorporeal Life Support Laboratory, Department of Surgery, University of Michigan, Ann Arbor, MI 48109, USA; zhaandre@umich.edu (A.Z.); alvaror@med.umich.edu (A.R.-P.); robbar@med.umich.edu (R.H.B.); 2 VA Ann Arbor Healthcare System, Ann Arbor, MI 48105, USA

**Keywords:** Extracorporeal CO_2_ Removal (ECCO_2_R), Extracorporeal Membrane Oxygenation (ECMO), ambulatory ECMO, Chronic Obstructive Pulmonary Disease (COPD), end-stage lung disease, wearable medical devices, negative feedback systems, smart ECMO

## Abstract

Extracorporeal Carbon Dioxide Removal (ECCO_2_R) systems support patients with severe respiratory failure. Concurrent ambulation and physical therapy improve patient outcomes, but these procedures are limited by the complexity and size of the extracorporeal systems and rapid changes in patient metabolism and the acid–base balance. Here, we present the first prototype of a wearable ECCO_2_R system capable of adjusting to a patient’s changing metabolic needs. Exhaust gas CO_2_ (EGCO_2_) partial pressure is used as an analog for blood CO_2_ partial pressure (pCO_2_). Twin blowers modulate sweep gas through the AL to achieve a desired target EGCO_2_. The integrated system was tested in vitro for 24 h with water, under varying simulated metabolic conditions and target EGCO_2_ values, and in a single test with whole blood. When challenged with changing inlet water pCO_2_ levels in in vitro tests, the system adjusted the sweep gas to achieve target EGCO_2_ within 1 min. Control runs with a fixed sweep gas (without negative feedback) demonstrated higher EGCO_2_ levels when challenged with higher water flow rates. A single in vitro test with whole ovine blood confirmed functionality in blood. This is the first step toward wearable ECCO_2_R systems that automatically respond to changing metabolism. Such devices would facilitate physical therapy and grant greater autonomy to patients.

## 1. Introduction

Chronic lung disease causes serious, long-term disability and is the fourth leading cause of death in the United States, responsible for over 150,000 deaths each year [1,2,3]. Acute respiratory distress syndrome (ARDS) has a 30–40% mortality rate and affects 190,000 Americans each year, most recently in patients with COVID-19 [4,5]. Mechanical ventilation alleviates some of the symptoms but can exacerbate the original illness and is limited by the failing lung [6]. Artificial-Lung (AL)-based treatments, such as Extracorporeal Membrane Oxygenation (ECMO) and Extracorporeal Carbon Dioxide Removal (ECCO_2_R), provide respiratory support without the drawbacks of mechanical ventilation-induced injury [7,8,9,10,11,12,13]. During acute cases, ALs allow the lung to rest and heal [9,14], and in chronic cases, ALs act as a bridge-to-transplant [10,11,15].

**Figure 1:**
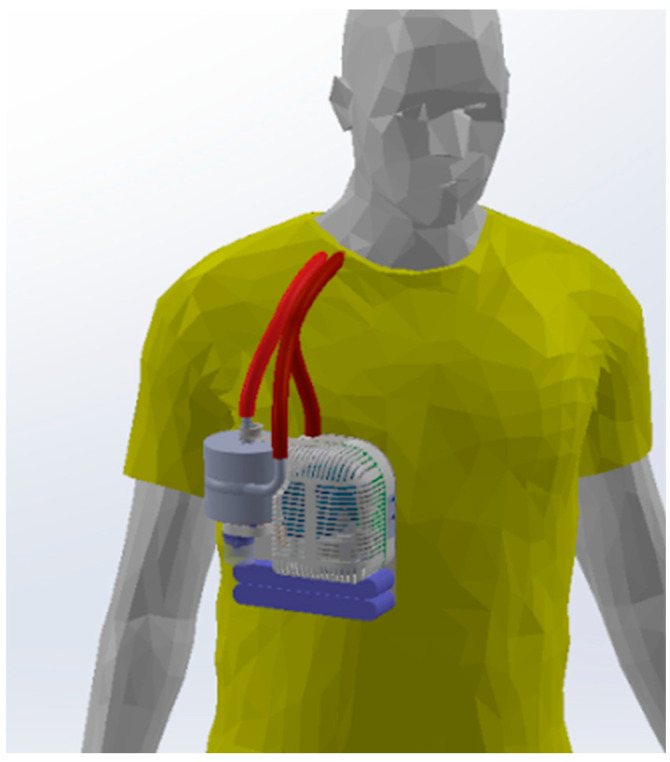
A wearable ECCO_2_R system would greatly simplify the logistics involved in physical therapy and patient transport, potentially enabling the patient to return home.

To increase patient mobility and thus improve the ability to exercise and rehabilitate, ambulatory ECMO systems have been developed in which the AL, blood pump, computer, battery, and oxygen cylinder are mounted to a wheeled pole [11,15,16]. By enabling ambulation and physical therapy while on ECMO, these systems significantly improve patient quality of life and survival compared to traditional non-ambulatory ECMO [17,18,19,20], while reducing the total hospital cost [21]. However, these extracorporeal systems are complex and bulky, limiting physical therapy [22,23,24]. Wearable systems under development by multiple research groups could potentially address these limitations (these systems are discussed under “Tradeoffs in Physical Size” in the discussions section) [25,26,27]. A concept diagram of a wearable ECCO_2_R system under development in our laboratory is shown in Figure 1.

Nevertheless, no current systems can automatically adjust to the changing metabolic needs of the patient. Activities that increase the metabolic rate or exacerbate the disease will cause respiratory acidosis unless the system is manually adjusted. Physical therapy in particular causes rapid changes in metabolic activity. Resting or healing of the disease will likewise cause respiratory alkalosis unless the system is manually adjusted. Adjustments to these factors are limited by the availability of hospital staff, forcing a tradeoff between cost and patient health and comfort. Additionally, adjustments require periodic blood draws and analysis, causing delays between measurement of the patient status and a change in treatment. Further, it removes the patient autonomy that would otherwise be granted by a portable or wearable system. Automatic controllers have been previously investigated, but all have included components that preclude use in a long-term ambulatory application [28,29,30,31,32,33,34,35,36,37].

Previous work by our laboratory has demonstrated that CO_2_ removal in an AL is a strong function of sweep gas flow [11,38]. Using this information, we developed a benchtop, laptop-controlled system that modulated the sweep gas flow rate to respond automatically and rapidly to simulated metabolic changes [39]. Subsequent work integrated all components into a battery-powered, portable package and improved overall performance metrics [25,40,41]. Here, we describe the next generation of that work: a further miniaturized, wearable, fully integrated, ruggedized ECCO_2_R system. A subset of this manuscript was presented as a conference abstract at the 2023 ASAIO Conference [42].

## 2. Materials and Methods

### 2.1. Engineering Design

The design of the wearable ECCO_2_R system is driven by the use cases of physical rehabilitation and ambulation.

These applications require a small and light form factor for wearable use, smaller than any other ECLS system reported (discussed in “Tradeoffs in Physical Size”). Specifically, it should be lighter than 2.0 kg and fit within a 25 × 25 × 10 cm footprint on the front of the chest. The system must be battery-powered, with a minimum 2 h of battery life to power the system for the duration of most ambulation activities. It must be ruggedized so it can continue operating after being dropped or bumped, as well as withstand minor amounts of debris. Critical components that are known to fail (battery and blowers) must be redundant.

The ability to automatically and quickly respond to changing patient blood CO_2_ levels would further increase patient comfort and the ability to exercise. It must be able to maintain a stable blood pCO_2_ when challenged with changing metabolic rates, particularly when compared to the current standard of care of a fixed sweep gas flow rate. To accomplish this, it should be able to maintain the partial pressure of Exhaust Gas CO_2_ (EGCO_2_) at a fixed value. This value should be achieved within 2 min and held steady within a range of ±5 mmHg and may be as low as 10 mmHg, which has been shown in previous studies to remove most of the CO_2_ in the blood [25,40,41]. The result is that the sweep gas flow rate should be higher during periods of high metabolism and lower during low metabolism.

Since this is an ECCO_2_R system as opposed to an ECMO system, it only needs to be able to process a fraction (1 L/min) of cardiac output. The system should be applicable in an arterio-venous attachment configuration or in veno-venous (VV) or veno-arterial (VA) with the addition of an external blood pump.

#### 2.1.1. Negative Feedback Control of EGCO_2_

**Figure 2:**
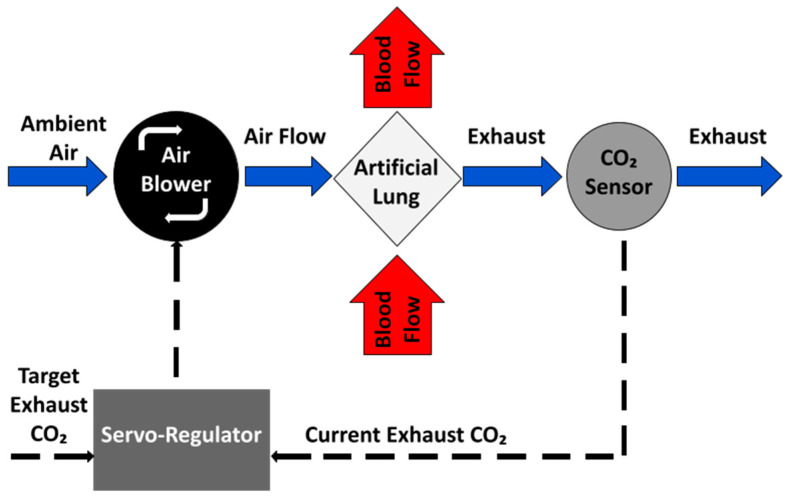
Conceptual overview of negative feedback using sweep gas to control EGCO_2_ in an AL.

To be able to respond to the changing respiratory needs of the patient, the system implements a negative feedback control loop, which automatically adjusts sweep gas flow rate through the AL (and thus CO_2_ removal) as required by the patient (Figure 2). Ideally, patient blood CO_2_ would be directly measured and used as the control signal in the negative feedback loop. However, a long-term blood-contacting CO_2_ sensor does not exist. Here, EGCO_2_ is used as a surrogate for blood CO_2_ and controlled using a proportional-integral-controller. This is described in more detail in previous studies [25,40,41]. In this process (Figure 2), the current EGCO_2_ value is read and compared to the target value (tEGCO_2_). If the EGCO_2_ is larger than the tEGCO_2_, sweep gas flow through the AL is increased to remove more CO_2_ from the blood passing through the AL, which in turn results in a lower EGCO_2_. If the current EGCO_2_ is less than the tEGCO_2_, then the sweep gas is decreased and less CO_2_ is removed from the blood.

The result is that the system can operate in a “fixed EGCO_2_” mode. How well the system is able to maintain a “fixed EGCO_2_” can be quantified by measuring settling time after a sudden metabolic change and the standard deviation in EGCO_2_ after settling.

#### 2.1.2. Miniaturization and Overall Design

**Figure 3:**
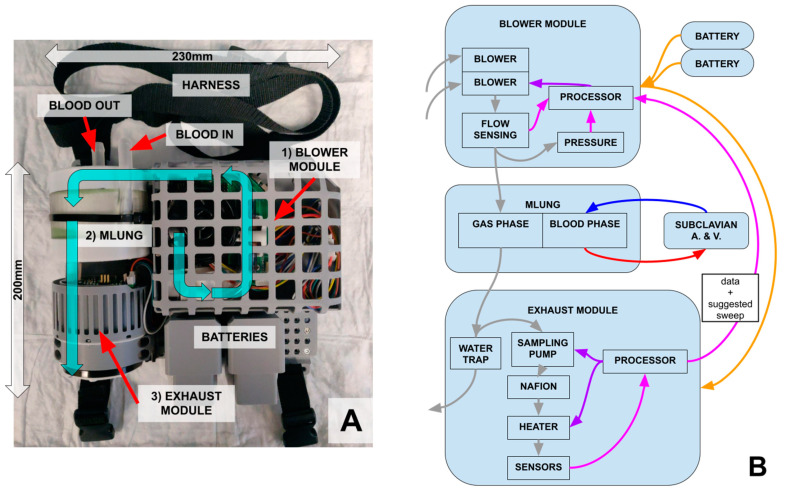
(**A**) Assembled and packaged prototype of the wearable ECCO_2_R system. Air (cyan arrows) is taken in from the blower module (1), pushed through the AL (2, MLung) where it removes CO_2_ from the blood, and then passes through the exhaust module (3), which reads the EGCO_2_. (**B**) Communication and control between system and subsystems. Gas flow is shown in gray, blood flow in red and blue, control in purple, power in orange, and data in pink.

A block diagram of the wearable ECCO_2_R system is shown in Figure 3. The main components are the blower module, responsible for setting the sweep gas (room air) flow rate, the artificial lung (i.e., the “MLung”), and the exhaust module, responsible for sensing CO_2_ and managing the negative feedback control loop.

To maximize comfort and mobility, a “pumpless sport-model” ECCO_2_R system was chosen [38,43]. This system accesses the subclavian artery and vein to maximize ambulation and uses an arteriovenous (AV) configuration to avoid the use of a pump, both making the device smaller and more compact and avoiding pump-related lysing [44,45]. As a tradeoff, O_2_ exchange is limited and it is not usable for patients that also require cardiac support.

To achieve a wearable form factor, the device must be designed around a specific AL. No other part of the system comes in contact with blood, and so, thrombosis and the immune response are almost entirely determined by the AL. The wearable ECCO_2_R system is designed around the MLung, which is being concurrently developed and tested under another project [46,47]. It is an AL optimized for CO_2_ removal in ESLD, making it ideal for the wearable system. It has a unique gated concentric design, allowing it to achieve similar CO_2_ removal as the Novalung iLA (Xenios, Heilbronn, Germany) while using a much lower fiber surface area (0.28 vs. 1.3 m^2^), thereby minimizing the stagnant flow, transit time, and thrombosis. The wearable system is compatible with the multiple versions of the MLung, which have varying pressure drop, blood flow capacity, and CO_2_ removal capability.

#### 2.1.3. Blower Module

**Figure 4:**
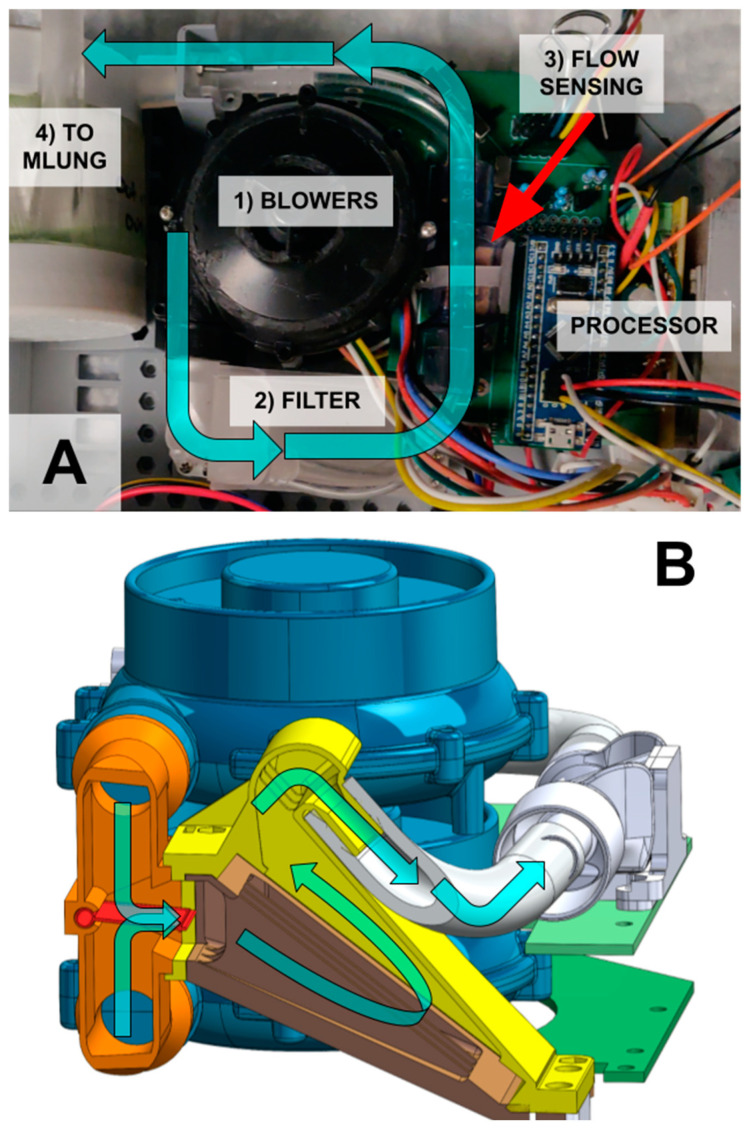
(**A**) The blower module takes in ambient air (cyan arrow) via two redundant, stacked blowers (1). The blowers push air through a bacterial filter (2), a flow sensor (3) and then into the AL (4). (**B**) The two blowers (blue) blow into a manifold (orange) with a moving valve (red). The valve allows flow from both blowers if they are used together but prevents backflow into the disabled blower if only one blower is on. To save space, this valve feeds directly into the filter housing (yellow). The filter can be replaced from the bottom (brown).

The blower module handles all safety critical processes, primarily controlling the flow of the sweep gas through the MLung. To achieve this, centrifugal WM-7040 blowers (Wei Cheng Electronics, Fenghua, China) push the ambient air sweep gas through a flow manifold and replaceable bacterial filter (Figure 4B) and into the AL. A flow sensor (Sensirion SFM4100, Stäfa, Switzerland) reads the sweep gas flow rate value. The blower module also has two ABP2 pressure sensors (Honeywell, Charlotte, NC, USA) to measure ambient pressure and the gas input pressure to control/prevent gas embolisms (i.e., gas bubbles in blood) due to high sweep gas pressures. These components are integrated and controlled by a low-power STM32F103C8T6 microprocessor (STMicroelectronics, Geneva, Switzerland).

Using feedback from the flow sensor, the blowers can be commanded to operate at a specific flow rate, allowing the blower module to act as an independent unit capable of operating in a fixed-sweep mode like existing clinical AL systems, if desired. If the blower module detects an error in the exhaust module, or if an error is indicated by the patient through a switch, it can resort to a pre-specified default fixed-sweep flow rate as in current standard of care systems. The supply of sweep gas to the AL is the highest priority (i.e., most safety critical) function of the system. For this reason, two redundant blowers and two redundant batteries were used to minimize the chance of failure of this safety critical function.

By using centrifugal blowers instead of compressed air, the system avoids the need for compressed gas tanks. This makes the system significantly more compact, and capable of being integrated into a truly wearable form factor. However, this also restricts the sweep gas to filtered room air instead of compressed O_2_. Although the system can still provide some supplementary oxygenation, it is better suited to ECCO_2_R applications.

With the controller on, the blower module is programmed to have a minimum flow rate of 250 mL/min, so that some air is always moving through the AL into the exhaust module. This serves two purposes. First, if flow is permitted to approach zero, the exhaust module will only measure stagnant air, rather than the air passing through the AL, and the negative feedback loop would not function correctly. Second, at low flow rates, the amount of time for the gas to flow through the AL to the exhaust module is long, increasing delay in the control loop and reducing the ability of the system to respond rapidly to changing blood CO_2_ levels.

#### 2.1.4. Exhaust (Smart) Module

The exhaust module handles all non-safety-critical processes, primarily reading and interpreting the EGCO_2_ and calculating the next suggested sweep gas flow rate. To that end, it has a sensing chamber containing a Sensirion STC31 CO_2_ sensor. Preceding the CO_2_ sensor is a water trap, Nafion™ tubing (CO2Meter TUB-0003, Ormond Beach, FL, USA), and a heated enclosure (40 °C) to prevent condensate from reaching the CO_2_ sensor, confirmed by a Sensirion SHTC3 humidity sensor. To reduce the volume of air that needs dehumidification, side-stream sampling is performed using a Thompson 2002–0215 sampling pump (USA Thompson, Sheboygan, WI, USA). A Honeywell ABP2 pressure sensor in the sensing chamber permits normalization of the CO_2_ measurement. As a precautionary measure, the exhaust gas flows through a bacterial filter at the outlet of the exhaust module. These exhaust module components are integrated with and controlled by a second STM32F103C8T6 microcontroller, which also communicates with the blower module.

**Figure 5:**
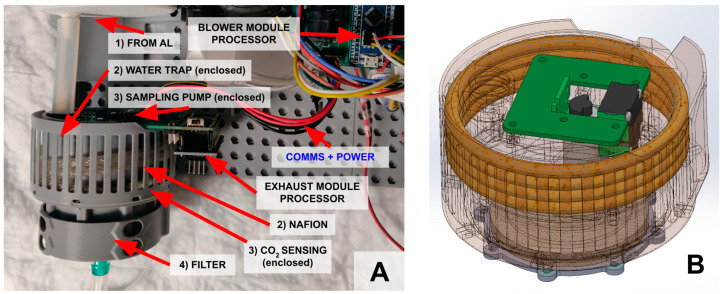
(**A**) After exiting the AL (1) the air flows into the exhaust module. The exhaust module consists of a water trap, Nafion tubing (2), and a sensing chamber (3, enclosed) and finally another bacterial filter (4). (**B**) A computer drawing of the sensor module. The sensing chamber and air channels are integrated into the chassis, and the remaining volume is used as the water trap. This maximizes space utilization of the available volume underneath the MLung.

Physically, the overall shape of the exhaust module is 80 mm in diameter to fit under the MLung. To achieve this, the water trap is integrated into the chassis (Figure 5B) and occupies any space not occupied by the sensing system, maximizing the use of space.

In addition to these physical components, the exhaust module performs the calculations for the negative feedback loop and “suggests” a sweep flow to the blower module. The blower module can ignore this “suggestion” if it detects something wrong with the exhaust module and default to the fixed-sweep mode. Some potential faulty behaviors could include the following: an unchanging suggested flow rate indicating a non-responsive exhaust gas module, or an unusually high, low, or unchanging reported EGCO_2_ value or suggested sweep gas value. As such, the exhaust module with its many sensors and negative feedback algorithm can fail without catastrophic consequences for the patient.

#### 2.1.5. Usability Features

The two redundant battery connections were implemented to be hot-swappable, so that a near-empty battery can be swapped with a full battery while the system continues being powered by the remaining battery. This hot-swappable design allows the batteries to be charged separately from the system, minimizing the risk from battery failure while charging and exposure to wall power. Battery power is sufficient for 3 h, although larger batteries can be installed if the patient is stationary.

**Figure 6:**
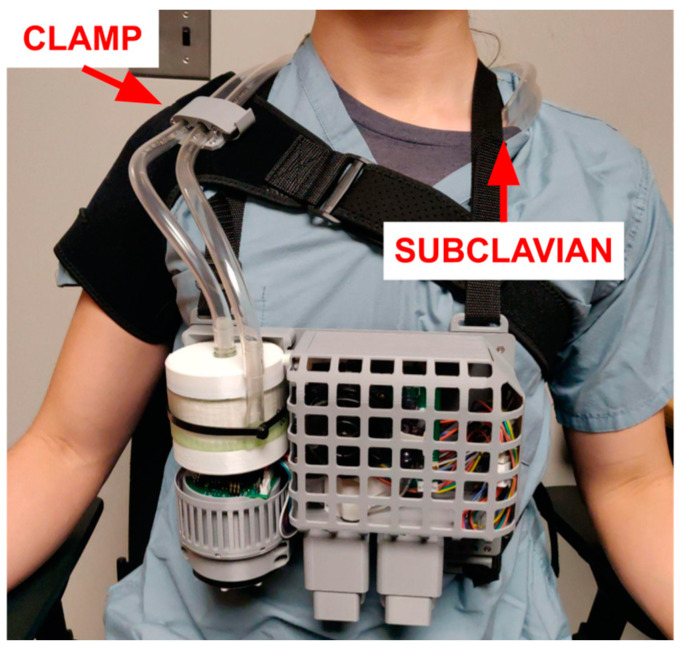
All components mounted onto the harness. The adjustable backplate can be worn by a human, sheep, and other similar-sized animals. The backplate also provides some impact resistance from drops. When the patient is lying in bed, the device should be removed and placed on the bed frame for patient comfort and to avoid bedsores.

All physical components are mounted onto a backplate with adjustable mounting points for a harness (Figure 6). The assembled device measures 23 × 20 × 8 cm and weighs 1.7 kg with a short version of the MLung [48] and 2.0 kg with a tall version of the MLung [49]. When a patient is lying down, the harness should be removed and placed on the bed railing to avoid bedsores.

In addition to the harness carrying the ECCO_2_R control system and AL, a separate “shoulder brace” (modified from [50,51]) is provided. This brace clamps to the tubing and absorbs forces that would cause decannulation or cause other strain on the cannula. Such a force may be caused by either dropping the system or the tubing being caught, with both possible scenarios in an ambulatory application. In addition, the brace keeps the blood tubing close to the patient, minimizing the risk of these events.

**Figure 7:**
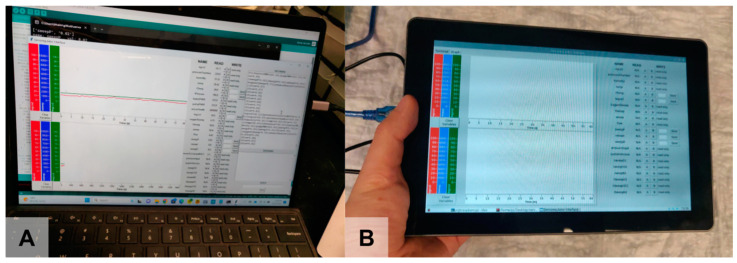
GUI running (**A**) on a laptop (full interface) and (**B**) on a tablet (compressed interface).

A basic laptop-based or tablet-based graphical user interface (GUI) and independent data logger assist with configuring the system and data acquisition during testing (Figure 7).

### 2.2. Subsystem Testing

The control of sweep flow and CO_2_ sensing were first tested individually. The blower module was attached to the tall MLung and commanded to maintain a set of target flows. Measured flow was compared to the target flow rate.

Next, the exhaust module was directly fed known mixtures of compressed CO_2_ and N_2_ to ensure it could measure a range of CO_2_ concentrations. Gas samples were manually taken from before the exhaust module and measured using a ABL800 Flex (Radiometer America, Brea, CA, USA). The ABL800 measurements were used as a benchmark to calibrate the exhaust module’s CO_2_ readings using a one-point calibration at 20 mmHg. It was then used to evaluate the accuracy of the exhaust module’s readings after calibration.

### 2.3. In Vitro Benchtop Testing with Water

**Figure 8:**
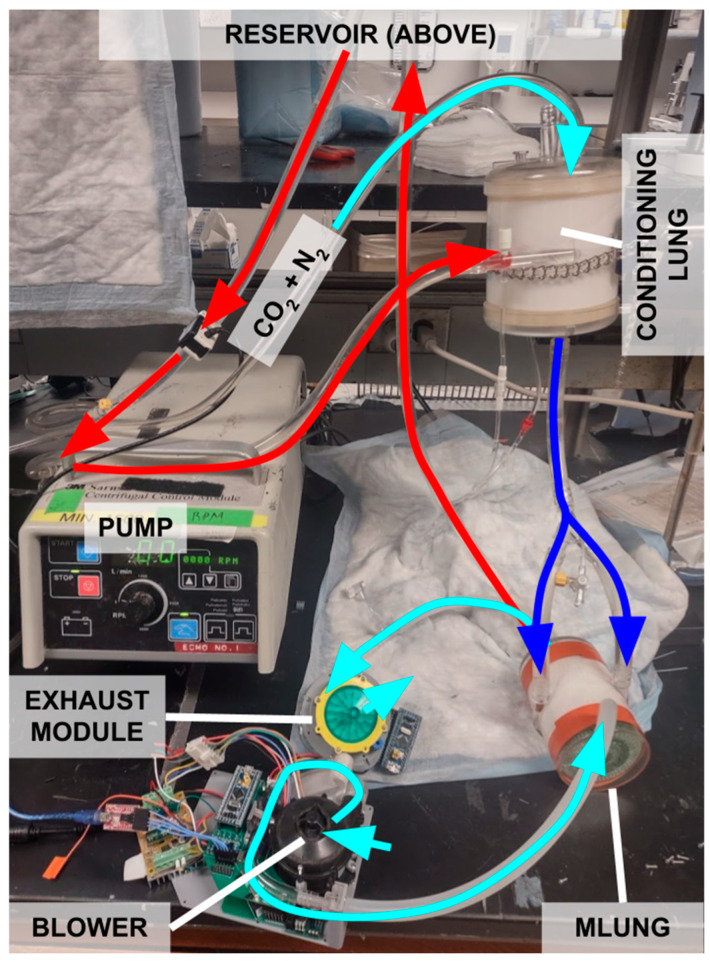
In vitro testing setup. Light blue arrows denote gas flows, and red and dark blue arrows denote water flows (water is used as an analog for blood). In this test, compressed CO_2_ was fed into the conditioning lung. The CO_2_ was dissolved into the circulating water by the conditioning lung. It was then removed by the MLung. After the MLung, the gas flow was routed to the exhaust module for EGCO_2_ sensing.

The wearable ECCO_2_R system was tested in vitro by using it to remove CO_2_ from water, an analog for how it would remove CO_2_ from blood in the clinical setting. It was challenged with a range of simulated metabolic conditions via changes in incoming water dissolved CO_2_ levels. The test circuit is pictured in Figure 8, showing all components of the wearable system, but deconstructed for initial testing.

The reservoir contained 5 gallons of deionized water, which is a suitable substitute for blood when only evaluating CO_2_ exchange [52].

A Terumo Cardiovascular Capiox RX25 oxygenator (Terumo Cardiovascular, Ann Arbor, MI, USA) was used as a blood conditioning lung. Varying mixtures of pure CO_2_ and air were pumped through the gas side of the conditioning lung to add CO_2_ to the water in varying amounts to simulate changes in patient metabolic activity. Air flow to the conditioning lung was fixed at 2 L/min, and CO_2_ was pumped at 0.1 L/min, 0.2 L/min, 0.4 L/min, or 0.8 L/min. Each conditioning lung setting was fixed for 4 min to allow the controller to react to each simulated metabolic change.

A centrifugal pump (3M Sarns, Maplewood, MN, USA) capable of varying water flow acted as a surrogate for the heart. A TS410 flow meter and ME-9PXL flow probe (Transonic, Ithaca, NY, USA) were used to measure water flow rates.

First, the performance of the ECCO_2_R system was evaluated under different tEGCO_2_s. The water flow rate was fixed at 1.0 L/min, while the tEGCO_2_ varied among 10 mmHg, 20 mmHg, 30 mmHg, and 40 mmHg. For control experiments, the blower module was commanded to operate at a 1 L/min or 2 L/min fixed sweep gas flow to simulate possible sweep values that could be prescribed by a physician with a normal ECCO_2_R system without active feedback. Standard deviations of various measurements were used as a metric of the system’s ability to maintain a steady-state in against a changing simulated metabolic rate.

Second, the performance of the system was evaluated under different simulated blood flow rates. The liquid pump was set to 0.5 L/min, 1.0 L/min, 1.5 L/min, or 2.0 L/min, with tEGCO_2_ fixed at 20 mmHg. For these tests, the 0.8 L/min CO_2_ conditioning level was omitted for expediency. As in the previous test, the EGCO_2_ sensor was calibrated via ABL800 measurements prior to initiating the test. The CO_2_ content in water was not evaluated because the ABL800 did not provide accurate measurements of CO_2_ in water.

### 2.4. Blood Testing Method

In order to validate the water testing results and confirm their validity, a single test was performed using the in vitro testing setup shown in Figure 8 but using blood in place of water. The blood flow rate was set to 1.0 L/min, and tEGCO_2_ was set to 20 mmHg. Air flow to the conditioning lung was fixed at 2 L/min, and CO_2_ was supplied at 0.1 L/min, 0.2 L/min, or 0.4 L/min to simulate changes in the metabolic rate.

## 3. Results and Analysis

### 3.1. Subsystem Testing Results

**Figure 9:**
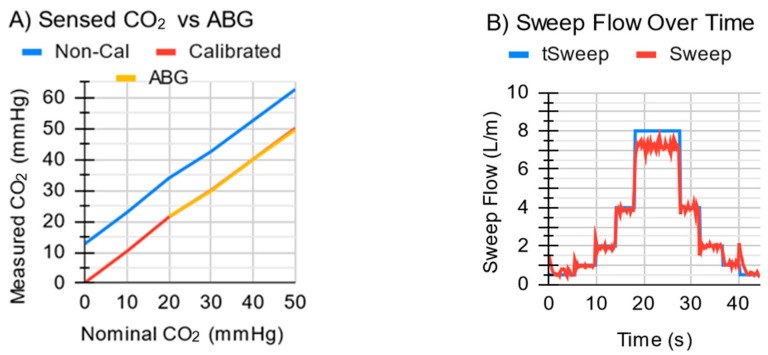
(**A**) The ECCO_2_R system measures CO_2_ in a gas stream, in reference to an arterial-blood-gas machine (ABG). With a 2-point calibration, the values are so close (<1 mmHg) that the lines almost overlap. (**B**) The system quickly attains desired sweep flows but exhibits a maximum sweep gas flow of ~7 L/min when tested with the tall MLung.

The ECCO_2_R system quickly (<2 s) attained a target sweep gas flow up to 4 L/min (Figure 9B) and exhibited a maximum sweep flow of 7 L/min with the tall MLung. Initial flow overshoot spikes were up to 0.5 L/min, but these quickly (<1 s) stabilized, at which point the error in the sweep flow averaged 0.09 L/min above the target, with a standard deviation of 0.08 L/min. The exhaust module measured EGCO_2_ for all values tested up to 50 mmHg with <1 mmHg error (Figure 9A) after calibration.

### 3.2. In Vitro Benchtop Testing with Water Results

**Figure 10:**
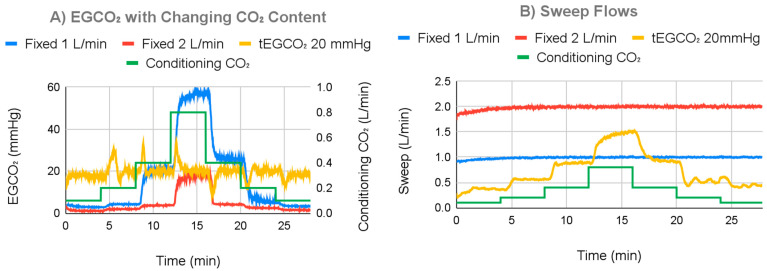
(**A**) EGCO_2_ changes based on simulated changing metabolism, when operating with negative feedback or with fixed sweep gas. (**B**) Sweep flow changes based on simulated changing metabolism when operating with negative feedback or with a fixed sweep gas.

The ECCO_2_R system demonstrated the ability to respond to simulated changes in patient metabolism to maintain a specified EGCO_2_ when the negative feedback system was in effect (Figure 10). This was in contrast to the 1.0 L/min and 2.0 L/min fixed sweep gas flow (current clinical standard of care) controls, where EGCO_2_ values varied widely as the simulated metabolic rate was changed. The settling time was consistently less than 1 min (Table 1). Figure 10A shows results for a 1.0 L/min water flow and a 20 mmHg tEGCO_2_. Over the entire testing time, average EGCO_2_ for a tEGCO_2_ of 20 mmHg was 19.43 mmHg with a 3.15 mmHg standard deviation. This is in contrast to that of a 1.0 L/min fixed sweep of 16.92 mmHg with a 17.80 mmHg standard deviation and a 2.0 L/min fixed sweep of 4.72 mmHg with a 5.40 mmHg standard deviation. After settling, the average EGCO_2_ with the controller on was 19.30 mmHg with a standard deviation of 1.27 mmHg. Overall, EGCO_2_ with the controller on varied between 9 and 34 mmHg. For a fixed sweep flow of 1.0 L/min or 2.0 L/min, EGCO_2_ varied between 3 and 58 mmHg or 1 and 20 mmHg, respectively (Table 2). Additional metadata for all tests are supplied in Supplementary Information Figure S1.

Figure 10B displays measured sweep gas flows for the experiment shown in Figure 10A. In Figure 10B for the fixed EGCO_2_ test (feedback on), sweep flow increased with increasing simulated metabolic rate and vice versa. When in fixed sweep mode, the ECCO_2_R system maintained the target sweep flow with gas flow standard deviations of 0.048 L/min at 1.0 L/min and 0.082 L/min at 2.0 L/min.

#### 3.2.1. Varying tEGCO_2_ Results

**Figure 11:**
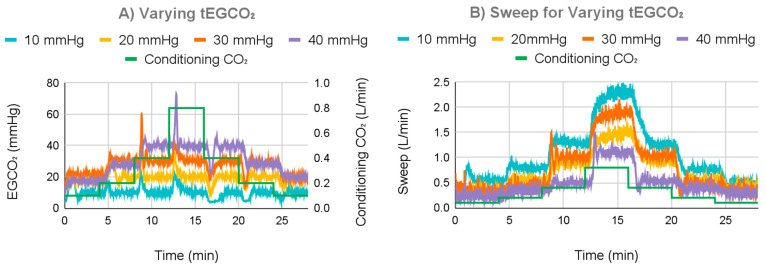
Measured EGCO_2_ (**A**) and sweep gas (**B**) under different tEGCO_2_ values.

For the next test, the control system was set to a tEGCO_2_ of either 10, 20, 30, or 40 mmHg, and incoming water CO_2_ content was varied to test the control system (Figure 11). For a tEGCO_2_ of 10 mmHg, the average EGCO_2_ was 9.92 mmHg with a 0.70 mmHg standard deviation once settled. For a tEGCO_2_ of 30 mmHg, at the lowest simulated metabolic rate, the measured EGCO_2_ was less than the tEGCO_2_ at the system’s minimum sweep gas flow rate, which averaged 0.35 L/min, and thus, the tEGCO_2_ could not be achieved. For the other simulated metabolic rates, the average EGCO_2_ was 29.97 mmHg with a 0.97 mmHg standard deviation. For a tEGCO_2_ of 40 mmHg, tEGCO_2_ could not be achieved for the smallest two simulated metabolic rates; the minimum sweep averaged 0.32 L/min. For the other metabolic rates, the average EGCO_2_ was 39.92 mmHg with a 0.90 mmHg standard deviation.

Average overshoot and undershoot were lowest at 7.99 mmHg for a tEGCO_2_ of 10 mmHg with a 3.24 mmHg standard deviation, increasing to 10.61 mmHg with a 2.91 mmHg standard deviation at 20 mmHg, 17.88 mmHg with a 8.45 mmHg standard deviation at 30 mmHg, and finally 25.85 mmHg with a 10.14 mmHg standard deviation at 40 mmHg.

#### 3.2.2. Varying Water Flow Rate Results

**Figure 12:**
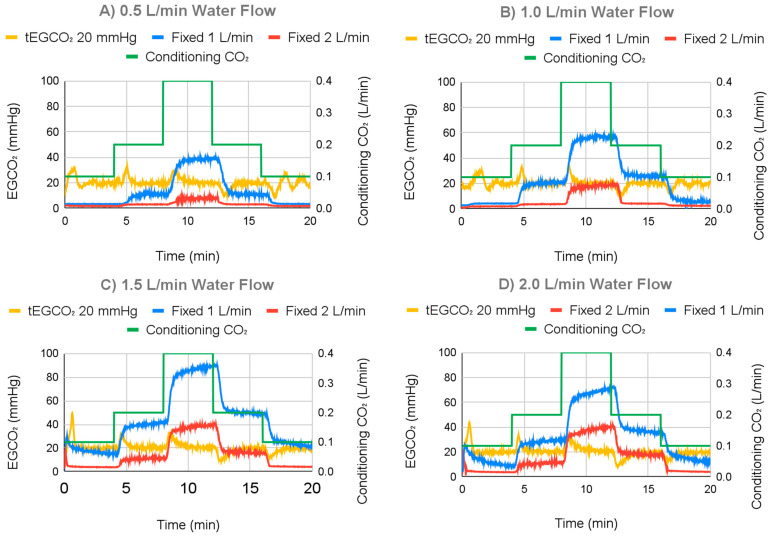
Individual graphs for varying water flow rates for 20 mmHg at (**A**) 0.5 L/min, (**B**) 1.0 L/min, (**C**) 1.5 L/min, and (**D**) 2.0 L/min.

The response of the ECCO_2_R sweep gas control system to changing water flow rates is displayed in Figure 12, demonstrating the ability to maintain a tEGCO_2_ of 20 mmHg across a range of flows. For the range of the water flow test, average EGCO_2_ with the controller on (fixed EGCO_2_) ranged from 20.19 mmHg to 20.41 mmHg, with standard deviations from 3.36 mmHg to 4.50 mmHg over the course of the run. In contrast, 1.0 L/min fixed sweep gas flow controls (controller off) had average EGCO_2_ values ranging from 13.24 mmHg to 43.39 mmHg, with standard deviations ranging from 12.61 mmHg to 24.30 mmHg. The 2.0 L/min fixed sweep gas flow controls had average EGCO_2_ values ranging from 3.43 mmHg to 15.09 mmHg, with standard deviations ranging from 2.02 mmHg to 12.34 mmHg. After settling, for water flows of 0.5 L/min, 1.0 L/min, 1.5 L/min, and 2.0 L/min and a fixed EGCO_2_ of 20 mmHg, average EGCO_2_ was 20.05 mmHg with a 1.59 mmHg standard deviation, 19.79 mmHg with a 1.09 mmHg standard deviation, 20.02 mmHg with a 0.75 mmHg standard deviation, and 20.03 mmHg with a 0.90 mmHg standard deviation, respectively.

Overshoot and undershoot of EGCO_2_ when targeting fixed EGCO_2_ varied between 10.92 mmHg and 12.61 mmHg with standard deviation varying from 1.32 mmHg to 2.63 mmHg. Neither the average or standard deviation of overshoot and undershoot appeared to correlate with changing water flow rates.

### 3.3. In Vitro Benchtop Testing with Blood Results

**Figure 13:**
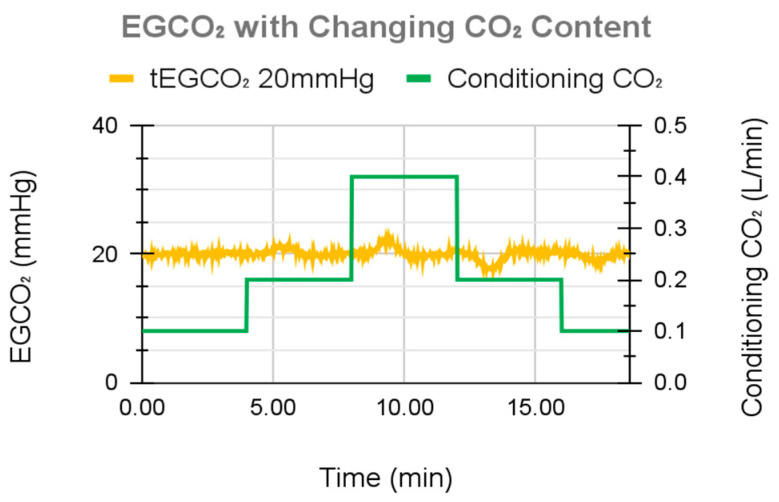
System performance during the blood flow test.

To validate the in vitro water experiments, a single test with whole blood was performed. tEGCO_2_ was set to 20 mmHg, and incoming blood CO_2_ was varied (by varying the concentration of CO_2_ in the sweep gas of the conditioning lung) to simulate changes in patient metabolic activity. The control system maintained the desired tEGCO_2_ under all simulated metabolic rates (Figure 13), demonstrating an EGCO_2_ maximum overshoot of 2.6 mmHg, maximum undershoot of 2.7 mmHg, and a standard deviation of 0.7 mmHg throughout the course of the study, including periods of changing metabolic rates.

## 4. Discussion

### 4.1. Analysis of Performance

Blower accuracy is 0.008 L/min when amortized over 10 s, although raw readings from the mass flow sensor were quite noisy at 0.25 L/min. The short MLung has half the gas-side resistance of the tall MLung and so is anticipated to have a maximum sweep of approximately 14 L/min.

Figure 12 demonstrates that the system is unable to add CO_2_ to blood and only remove it.

### 4.2. Mechanism of Action

Gas flow increased with an increasing simulated metabolic rate when operating in fixed EGCO_2_ mode. This allows the controller to maintain a fixed EGCO_2_ by increasing sweep gas flow during periods of high simulated metabolic rates to remove additional CO_2_ from the blood and vice versa.

It should be noted that as the gas flow rate increases, the time for pCO_2_ levels in the gas to equilibrate with pCO_2_ levels in the liquid decreases, and therefore, the difference between these two values is expected to increase as the sweep gas flow increases. Therefore, the effect of an increasing gas flow rate is expected to decrease the EGCO_2_ down to its targeted value but pCO_2_ in the blood or water to a lesser extent.

### 4.3. EGCO_2_ Overshoot during Simulated Metabolic Changes

The control system exhibited overshoot after each simulated metabolic increase in the in vitro water tests and undershoot after each simulated metabolic decrease. The over/undershoot varied between 7.99 mmHg at a tEGCO_2_ of 10 mmHg to 25.85 mmHg at 40 mmHg, which consistently settled to within 5 mmHg of the target value in less than 1 min. These overshoots were mitigated slightly by adjusting the negative feedback algorithm to be less aggressive. (Specifically, the proportional term of the proportional-integral-derivative controller was reduced, reducing overshoot at the cost of initial response time).

In the human body, blood completes a full cycle through the body in 10 to 120 s [53], approximately similar to the settling time of the control system. Further, changes in blood CO_2_ in humans are typically gradual and caused by rest/wake cycles, disease progression or healing, and changes in patient activity. The sudden step changes in incoming water and blood CO_2_ levels used in the in vitro tests were not fully realistic, but instead were employed to provide a difficult challenge for the control system and to ensure the control system did not resonate. Finally, any over/undershoot of tEGCO_2_ with the controller on was still significantly better than variation in the EGCO_2_ with the controller off (fixed sweep flow).

### 4.4. Inability to Perform Low CO_2_ Evacuation

While testing various tEGCO_2_ values (Figure 12), tEGCO_2_ settings of 30 and 40 mmHg could not meet the tEGCO_2_ value for the smallest simulated metabolic rates. In these instances, the measured EGCO_2_ was below the target value at the smallest permitted sweep gas flow rate. Since the sweep gas flow could not go lower, EGCO_2_ could not increase any more. In patients this should not be an issue, given that the system is meant for patients who have poor CO_2_ removal and thus high blood CO_2_ levels.

In fact, an extended period of fixed sweep flow in patients can sometimes lead to low blood CO_2_ levels (hypocarbia) and its associated complications (cerebral vasoconstriction, cardiac arrhythmia, and exacerbation of existing cardiorespiratory conditions). For a patient on a ventilator, hypocarbia can only be determined via periodic blood draws, and thus, detection (and an adjustment of sweep flow) may be delayed. The system described here should help prevent hypocarbia by automatically reducing the sweep gas flow when blood CO_2_ (and thus EGCO_2_) decreases.

### 4.5. Regulation of EGCO_2_ Rather than Blood CO_2_

In the presented wearable ECCO_2_R system, measured EGCO_2_ is used as the control signal for the control system. In this case, EGCO_2_ serves as a surrogate for directly measuring arterial blood pCO_2_ content (PaCO_2_), since no long-term blood CO_2_ sensor exists. However, being able to measure and regulate blood pCO_2_ directly would have been the superior choice, if possible.

EGCO_2_ is a function of both the blood pCO_2_ entering the AL and the sweep gas flow rate. Theoretically, a sufficiently low sweep gas flow rate would equilibrate gaseous CO_2_ levels with the blood pCO_2_, such that the EGCO_2_ and the post-AL blood pCO_2_ are effectively identical. As the sweep increases, the sweep gas has less time to equilibrate, and so there is a greater gap between post-AL blood pCO_2_ and EGCO_2_. In the context of this study, even though the controller maintains a constant EGCO_2_ value, the post-AL blood pCO_2_ likely exceeds the EGCO_2_. Further, higher metabolic rates result in higher sweep gas flow rates and therefore a higher post-AL blood pCO_2_. This relationship has been demonstrated in vitro [25] and in vivo [41] in previous studies that also used a fixed EGCO_2_ method.

Additionally, since this device is designed for subclavian AV ECCO_2_R, it processes a fraction (~10–20%) of the circulating blood. A sicker patient would have a higher pCO_2_ in the blood not processed by this device, and so the blood leaving this device would need to have lower pCO_2_ to compensate during remixing. Using the fixed EGCO_2_ method, such a patient would be prescribed a lower tEGCO_2_ than a healthier patient.

With that said, a fixed EGCO_2_ sweep gas control system is still superior to fixed sweep gas. Firstly, if a physician was present, they would prescribe increased sweep during periods of increased metabolism and less sweep during periods of decreased metabolism, which is exactly how the presented controller responds in Figure 11. Secondly, higher metabolic levels should result in a higher PaCO_2_ than lower metabolic levels, even with fixed EGCO_2_. Therefore, the system is undercompensating rather than overcompensating. In conclusion, the performance of the negative feedback system likely falls between a fixed sweep system and a theoretical system, which would monitor and directly control blood CO_2_ (such as a physician constantly taking blood-gas readings).

In practice, the rate at which a physician can change sweep is limited not only by their availability but also by the rate of blood draws, which a carry risk of infection and anemia.

We envision that a physician would prescribe a tEGCO_2_ based on the patient’s baseline PaCO_2_, with a lower tEGCO_2_ corresponding to sicker patients. The negative feedback system would then reduce PaCO_2_ variations during changes in activity and the disease state that take place in between blood draws and physician inspections.

### 4.6. Tradeoffs in Physical Size

This system is smaller by an order of magnitude than any other clinical ECMO or ECCO_2_R system to date. Other “wearable” ECMO systems only have the AL and blood pump (if it exists) in a wearable form factor but rely on a separate package to house the gas supply and control system [26,27,54,55,56].

When considering weight, the use of blowers using ambient air rather than compressed O_2_ had the most significant impact. Portable O_2_ concentrators weigh upwards of 800 g, and systems with higher flow rates weigh closer to 2 kg [57]. Portable O_2_ tanks for short-term ambulation weigh 1.36 kg and would last 2.75 h at 1 L/min [58]. These would have roughly doubled the weight of the system. In comparison, the two blowers used on the reported system weigh only 90 g each.

When considering space, the greatest impact was in the geometric packaging of all the components. In particular, the control electronics were built around the other components to maximize use of space and minimize overall size. However, this restricts the choice of AL to devices with similar dimensions to the MLung (without physical modifications to the system).

The sensor module weighs approximately 100 g and adds 80 mm to the bottom of the MLung. Although not negligible, its presence does not significantly impede wearability, while the advantage of negative feedback on CO_2_ removal addresses changing metabolism during physical rehabilitation.

A summary of advantages and disadvantages and comparisons with other ambulatory ECMO and ECCO_2_R systems is shown in Table 3.

### 4.7. Planned Future Studies

Future studies are planned in which these in vitro water studies are replicated in vitro with blood. During these blood studies, pre-AL and post-AL blood pCO_2_ values will be measured. It will then be tested in vivo in a sheep model.

## 5. Conclusions

A wearable ECCO_2_R system capable of automatically responding to changes in blood pCO_2_ levels has been described. Specifically, it is able to operate in a “fixed EGCO_2_” mode. Additionally, it is smaller and more portable than any other complete ECLS or ECCO_2_R system reported. It serves as the blueprint for a system that would allow ECCO_2_R patients to be treated with fewer resources, and better comfort, autonomy, and outcomes. In particular, it could reduce many of the logistical challenges associated with physical therapy while on ECCO_2_R.

## Figures and Tables

**Table 1:** Average, standard deviation, maximum, and minimum EGCO_2_ for each test, along with settling times for fixed EGCO_2_ tests. This includes periods of metabolic change.

Sweep Control	Avg EGCO_2_ (mmHg)	Stdev EGCO_2_ (mmHg)	Max EGCO_2_ (mmHg)	Min EGCO_2_ (mmHg)
10 mmHg	10.09	3.05	23.15	3.92
20 mmHg	19.43	3.15	34.38	9.24
30 mmHg	27.23	5.49	60.09	9.80
40 mmHg	30.22	9.80	73.02	9.80
1.0 L/min	16.92	17.80	57.85	2.73
2.0 L/min	4.72	5.40	20.20	0.85

**Table 2:** Settling time was judged as the time from when the EGCO_2_ diverged from the tEGCO_2_ by greater than 5 mmHg after a change in the metabolic rate, until it was within 5 mmHg for 3 s. Average, standard deviation, max, and minimum values are taken starting 120 s after a simulated metabolic change, to evaluate performance under a steady state. A more detailed chart is shown in Supplementary Information, Figure S1.

Sweep Control	Settling Time (min)	Avg EGCO_2_ (mmHg)	Stdev EGCO_2_ (mmHg)	Max EGCO_2_ (mmHg)	Min EGCO_2_ (mmHg)
10 mmHg	0.63	9.92	0.70	12.61	6.99
20 mmHg	0.51	19.30	1.27	22.16	14.72
30 mmHg	0.58	29.97	0.97	32.42	26.52
40 mmHg	0.53	39.92	0.90	42.53	37.48

**Table 3:** Comparison of the current system with similar ECMO and ECCO_2_R systems.

Feature	Wearable Smart ECCO_2_R	Portable Smart ECCO_2_R [25]	Breathe [26]	Wearable Pumping AL [27]
High FiO_2_	No	No	Yes	Yes
Compatible with multiple ALs	No	Yes	No	No
Wearable	Yes	No	With separate gas supply and controller	With separate gas supply and controller
Active negative feedback	Yes	Yes	No	No
Blood pump (for VA or VV operation)	No	Yes	Yes	Yes

## Data Availability

Computer models for the physical system, electrical schematics and models, and raw data from in vitro water and blood tests are available at the following repository: https://drive.google.com/open?id=1Xc-3LHa-zxE3eEoFRV539kPIU1JthFxM&usp=drive_fs (accessed on 19 September 2024). Code and algorithms are available at the following: https://github.com/azhang100/servo_wearable (accessed on 19 September 2024).

## Supplementary Materials

The following supporting information can be downloaded at: https://www.mdpi.com/article/10.3390/bioengineering11100969/s1, Table S1: Metadata for each run; Figure S1: Oxygen shroud; Table S2: Latency for each run.
