# Supplementary material for: A Wearable Extracorporeal CO2 Removal System with a Closed-Loop Feedback
Source: Bioengineering (Basel). 2024 Sep 27;11(10):969. doi: 10.3390/bioengineering11100969 (PMC11505386; doi:10.3390/bioengineering11100969)
Supplement: Supplementary file 1 [file bioengineering-11-00969-s001.zip › bioengineering-3175806-supplementary.pdf]

Supplementary

Table S1: Metadata for each run.

Sweep Control	Water Flow	Avg $EGCO_2$ (mmHg)	Stdev $EGCO_2$ (mmHg)	Max $EGCO_2$ (mmHg)	Min $EGCO_2$ (mmHg)	Settling Time (min)	Avg $EGCO_2$ after 120s (mmHg)	Stdev $EGCO_2$ after 120s (mmHg)	Max $EGCO_2$ after 120s (mmHg)	Min $EGCO_2$ after 120s (mmHg)	Avg Over-shoot / Under-shoot (mmHg)	Stdev Over-shoot / Under-shoot (mmHg)
10 mmHg	1.0 L/min *	10.09	3.05	23.15	3.92	0.63	9.92	0.70	12.61	6.99	7.99	3.24
20 mmHg	1.0 L/min *	19.43	3.15	34.38	9.24	0.51	19.30	1.27	22.16	14.72	10.61	2.91
30 mmHg **	1.0 L/min *	27.23	5.49	60.09	9.80	0.58	29.97	0.97	32.42	26.52	17.88	8.45
40 mmHg **	1.0 L/min *	30.22	9.80	73.02	9.80	0.53	39.92	0.90	42.53	37.48	25.85	10.14
1.0 L/min	1.0 L/min *	16.92	17.80	57.85	2.73							
2.0 L/min	1.0 L/min *	4.72	5.40	20.20	0.85							
20 mmHg	0.5 L/min	20.19	3.68	32.70	8.96	0.59	20.05	1.59	27.36	15.00	10.92	1.32
1.0 L/min	0.5 L/min	13.24	12.61	40.29	3.15							
2.0 L/min	0.5 L/min	3.43	2.02	9.24	1.58							
20 mmHg	1.0 L/min	20.09	3.36	34.38	9.24	0.48	19.79	1.09	22.16	14.72	11.73	2.63
1.0 L/min	1.0 L/min	22.32	18.46	57.85	2.73							
2.0 L/min	1.0 L/min	6.01	5.91	20.20	0.85							
20 mmHg	1.5 L/min	20.39	4.50	49.56	8.54	0.70	20.02	0.75	22.30	17.95	11.13	1.91
1.0 L/min	1.5 L/min	43.39	24.30	90.58	5.73							
2.0 L/min	1.5 L/min	14.67	12.26	40.57	3.57							
20 mmHg	2.0 L/min	20.41	4.40	43.80	7.69	0.74	20.03	0.90	22.86	17.53	12.61	1.82
1.0 L/min	2.0 L/min	31.96	19.87	72.74	4.40							
2.0 L/min	2.0 L/min	15.09	12.34	41.69	3.57							

* Runs include 0.8 L/min

** Could not stabilize at some metabolic rates

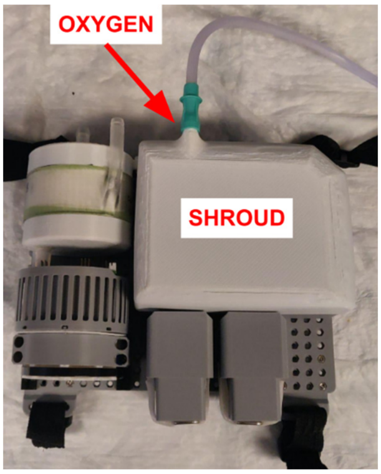

Figure S1: Oxygen shroud. An O_2 shroud can be placed over the blower modules. This causes the blowers to take in O_2 in place of ambient air, increasing O_2 provided to the patient. Keeping the shroud in place during physical therapy requires the O_2 supply to be carried separately, with the oxygen concentrator or tank being worn at the hip or on a movable pole. However, since the system is designed for AV ECMO, oxygenation potential is limited unless a blood pump is added for VV or AV operation.

Table S2: Latency for each run.

**Sweep Control**	20 mmHg	20 mmHg	20 mmHg	20 mmHg	10 mmHg	20 mmHg	30 mmHg **	40 mmHg **
**Water Flow**	0.5 L/min	1.0 L/min	1.5 L/min	2.0 L/min	1.0 L/min *	1.0 L/min *	1.0 L/min *	1.0 L/min *
Average Latency (s)	44.85	32.7	22.5	22.95	35.8	40.8	29.55	30.6
Stdev Latency (s)	3.65	6.53	3.12	10.33	9.62	14.81	6.03	2.55
95% Uncertainty (s)	7.15	12.79	6.11	20.24	18.86	29.03	11.81	4.99

* Runs include 0.8 L/min
** Could not stabilize at some metabolic rates

Total latency for each of the runs is shown in Table S2 and is quantified as the time between when the conditioning CO_2 flow rate was changed and when the $EGCO_2$ diverged from the $tEGCO_2$ by greater than 5 mmHg. It can be observed that latency is lower for faster blood flows and higher for slower blood flows. Latency across all 1.0 L/min runs was 35.6±20.0 s. This is believed to be caused by several components:

Firstly, the changing sweep gas concentration must arrive at the conditioning AL, and then, this must cause the blood pCO_2 in the conditioning AL to change. The time it takes for the sweep gas concentration to reach the AL is estimated to be minimal (1-2 s) because they are mixed just before entering the conditioning AL. The priming volume of the Capiox FX25 is 260 mL, which corresponds to 15.6 s of dwell time at 1 L/min.

First, blood travels from the area of metabolism (the conditioning AL in the *in vitro* test setup) to the AL (the test AL in the *in vivo* test setup). *In vitro*, this latency is minimal (1-2 s) because the conditioning lung is placed immediately before the test AL. This is intended to stress the system and does not represent the situation *in vivo*.

Second, the changing inlet blood pCO_2 must be reflected in the $EGCO_2$. This is dependent on the time it takes for the blood to fill the AL. The MLung priming volume is ~70 mL (4.2 s blood dwell time at 1.0 L/min blood flow) for the short MLung and ~170 mL (10.2 s blood dwell time at 1.0 L/min blood flow) for the tall MLung, which was used in the *in vitro* studies.

Finally, the change in the $EGCO_2$ is then delayed by the time it takes for gas to travel from the MLung to the CO_2 sensor. This value is estimated to be 6.3 s at minimum sweep and is a function of the gas flow rate and dead space volume. After the system detects a changed $EGCO_2$, it responds by changing the sweep gas flow rate in < 1 s.

Total latency will be measured in future *in vivo* studies. *In vivo*, the change is much more gradual, and metabolism is separated from the AL by the entire body, including the pulmonary system. Therefore, the latency between inlet blood pCO_2 and the sensed $EGCO_2$ values is not expected to play a significant role in the effectiveness of the $ECCO_2R$ system (unlike *in vitro* studies).
